# Developing community pharmacists’ role in the management of type 2 diabetes and related microvascular complications: a nationwide survey in Australia

**DOI:** 10.7717/peerj.14849

**Published:** 2023-02-16

**Authors:** Louise Woodhams, Leanne Chalmers, Graham S. Hillis, Tin Fei Sim

**Affiliations:** 1 Curtin Medical School, Curtin University, Perth, Western Australia, Australia; 2 Medical School, The University of Western Australia, Perth, Western Australia, Australia; 3 Department of Cardiology, Royal Perth Hospital, Perth, Western Australia, Australia

**Keywords:** Community pharmacy, Microvascular complications, Type 2 diabetes, Professional pharmacy services

## Abstract

**Background:**

Community pharmacists have regular interactions with people living with type 2 diabetes to supply medications, and have a potential role in supporting other primary care professionals in the screening, management, monitoring and facilitation of timely referral of microvascular complications. This study aimed to investigate the contemporary and future roles of community pharmacists in diabetes-related microvascular complication management.

**Methods:**

This study involved an online Australian nation-wide survey of pharmacists administered *via* Qualtrics® and distributed through social media platforms, state and national pharmacy organisations, and *via* major banner groups. Descriptive analyses were undertaken using SPSS.

**Results:**

Among 77 valid responses, 72% of pharmacists already provided blood pressure and blood glucose monitoring services for the management of type 2 diabetes. Only 14% reported providing specific microvascular complication services. Over 80% identified a need for a comprehensive microvascular complication monitoring and referral service, and agreed it is feasible and within the scope of practice of a pharmacist. Almost all respondents agreed that they would implement and provide a monitoring and referral service if provided with appropriate training and resources. Potential barriers to service implementation were competing demands and lack of remuneration and awareness among consumers and health professionals.

**Conclusions:**

Type 2 diabetes services in Australian community pharmacies do not currently focus on microvascular complication management. There appears to be strong support for implementing a novel screening, monitoring and referral service *via* community pharmacy to facilitate timely access to care. Successful implementation would require additional pharmacist training, and identification of efficient pathways for service integration and remuneration.

## Introduction

Type 2 diabetes is a chronic health condition with the risk of long-term microvascular complications. People with type 2 diabetes often live at home and hence access to primary healthcare is essential in the management of diabetes and its associated complications. These complications are responsible for significant morbidity and reduction in quality of life, and include diabetic kidney disease (DKD), formally known as diabetic nephropathy, as well as diabetic retinopathy and neuropathy (Gheith et al., 2016). These complications are insidious in their progression and challenging to monitor and manage, however the accessibility of pharmacists and their regular interactions with patients (Brewster et al., 2020), could potentially provide an opportunity for regular monitoring. Early detection of microvascular complications improves prognosis, quality of life, and the overall cost (Chapman et al., 2019), especially if patients are referred to specialists and their conditions managed before progressing to severe disease states. Primary care professionals such as pharmacists could potentially be involved in the screening and monitoring of microvascular complications associated with type 2 diabetes to assist in early detection and referral to appropriate health professionals.

Contemporary models of service delivery already exist internationally and it is important to evaluate the need for this service in Australia (Dewsbury, Rodgers & Krska, 2015; Soprovich et al., 2019). Established and successful diabetes-related pharmacy services from countries such as the United Kingdom, Canada and the United States of America provide useful insight in the implementation of an outcome-focused patient-centred diabetes service (Dewsbury, Rodgers & Krska, 2015; Soprovich et al., 2019; Pfleger et al., 2008; Pousinho et al., 2016; Wibowo et al., 2015; Yaghoubi et al., 2017). The incorporation of monitoring and referral services for microvascular complications in community pharmacies has been shown to significantly improve clinical outcomes. The most notable services incorporated into international community pharmacies include pharmacist-led foot care interventions for people living with type 2 diabetes. These interventions ranged from promotion of foot examinations by podiatrists to improving foot self-care behaviours (Soprovich et al., 2019).

The existing diabetes-related programs in Australian community pharmacies do not currently focus on microvascular complication management (Pousinho et al., 2016). As it is a chronic health condition, people with type 2 diabetes spend most of their time living at home. This further highlights the importance of good access to primary healthcare services in the management of diabetes and its associated complications. Currently, community pharmacies can provide diabetes-specific services such as diabetes-related medication reviews and management programs (Krass et al., 2007). Whilst these services are efficient in monitoring clinical outcomes such as glycated haemoglobin (HbA1c), blood glucose level (BGL) and blood pressure, they do not address the gap in monitoring and referral for microvascular complications at the time of dispensing of a repeat prescription.

Effective screening and monitoring of microvascular complications is a core component in type 2 diabetes management (Medical Advisory Secretariat, 2009). The incorporation of new service delivery model or an extension to existing diabetes-related services in community pharmacies, to provide formal screening, monitoring and referral pathways to allied health professionals could be implemented (Sim et al., 2020). Moreover, pharmacists could act as a ‘bridge’ between patients and health professionals. Considering the increasing prevalence of diabetes (Cho et al., 2018) and hospitalisations following complications, there is an urgent need to develop innovative services, strategies and better employ the healthcare system for appropriate management of microvascular complications.

This study aimed to investigate the contemporary and future roles of pharmacists in microvascular complication management associated with type 2 diabetes in Australian community pharmacy. The specific objectives were to:

1. investigate the current practice of community pharmacists in diabetes management, specifically in screening, monitoring and management of microvascular complications,
2. explore community pharmacists’ knowledge, attitudes and perspectives on the implementation of microvascular complication management services and the required implementation strategies in community pharmacies,
3. identify the need for continued professional development and learning gaps to guide the development of future training, and
4. explore potential service delivery models which enable seamless follow-ups and referrals to health professionals.

It is anticipated that these investigations will provide the foundation for the development of a contemporary service delivery model that will improve the quality of life in people living with type 2 diabetes, reduce the economic burden, and streamline the management of type 2 diabetes in primary care. There is currently no published study investigating a novel screening, monitoring and formal referral service for microvascular complications associated with type 2 diabetes in Australian community pharmacies.

## Materials and Methods

### Ethics

Ethics approval was received from Curtin University Human Research Ethics Committee: Approval number HRE2021-0442.

### Study design

This was an exploratory study using a self-administered questionnaire administered online through Qualtrics®.

### Study population

All pharmacists practising in community pharmacy within Australia were eligible to complete the questionnaire. Pharmacists practising solely in hospital, research, academia or other practice settings were excluded from this study. Consent was implied by submission of a completed questionnaire.

### Sample size estimation

The Pharmacy Board of Australia registrant data showed 31,794 pharmacists were registered with the Australian Health Practitioner Regulation Agency in September 2020 (Pharmacy Board of Australia, 2020). In 2017, approximately 64.7% (Australian Government Department of Health, 2017) of pharmacists worked in community pharmacy, therefore the population size was approximately 20,571. A sample size of 378 was calculated and confirmed with the Qualtrics^®^ online sample size calculator.

### Study instrument—Questionnaire

The questionnaire (Material S1) was developed based on existing literature from international studies (Pfleger et al., 2008; Wibowo et al., 2015; Simpson et al., 2009). It included five sections with 81 questions. Part I: Current practice of community pharmacists in diabetes management (26 questions); Part II: Attitudes on microvascular complication management services (24 questions); Part III: Knowledge, continuing professional development and training (11 questions); Part IV: Potential service delivery models (eight questions); and Part V: Pharmacist demographic details (12 questions). Parts I to III were assessed through five point Likert scales from strongly disagree to strongly agree. Parts IV and V consisted of ranking questions. Free-text questions were available at the end of each section to capture any additional comments. The questionnaire was face and content validated by six academic pharmacists on computer and mobile devices. Their feedback was incorporated *via* minor adjustments to the questionnaire wording and format to improve clarity.

### Data collection and sampling methods

The questionnaire was available online *via* Qualtrics® and distributed through Facebook (between August and December 2021) and state and national pharmacy organisations’ electronic newsletters (between September and November 2021). Letters of invitation with the link and QR code to complete the questionnaire were also sent to 505 community pharmacies across Australia from three large banner groups in October 2021. These banner groups were selected based on number of pharmacies in the group and feasibility of sending letters of invitation. To meet the study objectives, a focus was placed on recruiting banner groups that offer professional services. At the completion of the questionnaire, participants were invited to enter a prize draw to win one of three $50 gift cards.

### Data analysis and synthesis

Results from the questionnaire were downloaded into SPSS V28.0.1.1 for analysis. Questionnaires that were not completed beyond the participant information and consent form were excluded from the analysis. Submitted responses with partial or complete data were included. Where reporting on questions where data was missing, the number of actual responses to the question was used as the denominator.

### Statistics

Descriptive statistics were reported (means and standard deviations for continuous data, and frequency and percentage for categorical data).

## Results

### Demographic data

Of the 109 responses received, 32 were excluded from the analysis because the questionnaires were not completed beyond the participant information and consent form. A total of 77 questionnaire responses were included in the analysis. The response rate could not be determined due to the distribution of the questionnaire link and QR code on social media platforms, pharmaceutical organisations and electronic invitation letters sent directly to community pharmacies. The demographic data of the participants and characteristics of their respective community pharmacies are reported in Table 1.

**Table 1:** Demographic data of participants and respective community pharmacy characteristics (*n* = 77).

Pharmacist data (*n* (%))	
Gender	
Male	20 (33)
Female	39 (64)
Prefer not to say	2 (3)
Age (years)	
<30	34 (55)
30–40	19 (31)
41–50	5 (8)
51–60	4 (6)
Number of years registered as a pharmacist	
<5	35 (56)
5–10	9 (15)
11–15	11 (18)
16–20	5 (8)
>20	2 (3)
Highest qualification in pharmacy	
Bachelor of pharmacy	35 (56.5)
Bachelor of pharmacy (Hons)	18 (29)
Master of pharmacy	9 (14.5)
Accredited pharmacist	
Yes, accredited pharmacist	15 (25)
Yes, undergoing accreditation	8 (13)
No, not an accredited or undergoing accreditation	38 (62)
Accredited diabetes educator	
Yes, accredited diabetes educator	2 (3)
No, but undergoing accreditation	1 (2)
No, not an accredited diabetes educator or undergoing accredited	58 (95)
Primary role in Pharmacy	
Sole proprietor	3 (5)
Partner proprietor	3 (5)
Pharmacist in charge	17 (27)
Employee pharmacist	35 (56.5)
Other	4 (6.5)

Pharmacy data (*n* (%))	
State/territory	
Australian Capital Territory (ACT)	1 (2)
New South Wales (NSW)	7 (11)
Northern Territory (NT)	1 (2)
Queensland (QLD)	7 (11)
South Australia (SA)	4 (6.5)
Tasmania (TAS)	0 (0)
Victoria (VIC)	4 (6.5)
Western Australia (WA)	38 (61)
Pharmacy location	
Shopping strip	17 (27)
Shopping centre	26 (42)
Isolated	6 (10)
Next to medical centre	11 (18)
Other	2 (3)
Location	
City	6 (10)
Suburb	46 (74)
Rural	9 (14)
Remote	1 (2)
Pharmacy location with respect to medical centre	
Co-located	16 (26)
<100 m	27 (44)
101–500 m	8 (13)
501 m–1 km	6 (10)
>1 km	4 (7)
Pharmacy size (Annual turnover)	
Small <$2 million/annum	14 (22)
Large >$2 million/annum	29 (47)
Unsure	19 (31)

**Note:**

Suburb is an area within a metropolitan area that is primarily a residential area. Rural and remote Australia are all areas outside Australia’s major cities. Rural is inner and outer regional areas. Remote areas are beyond outer regional areas.

### Current practice of community pharmacists in diabetes management, specifically in screening, monitoring and management of microvascular complications

**Figure 1:**
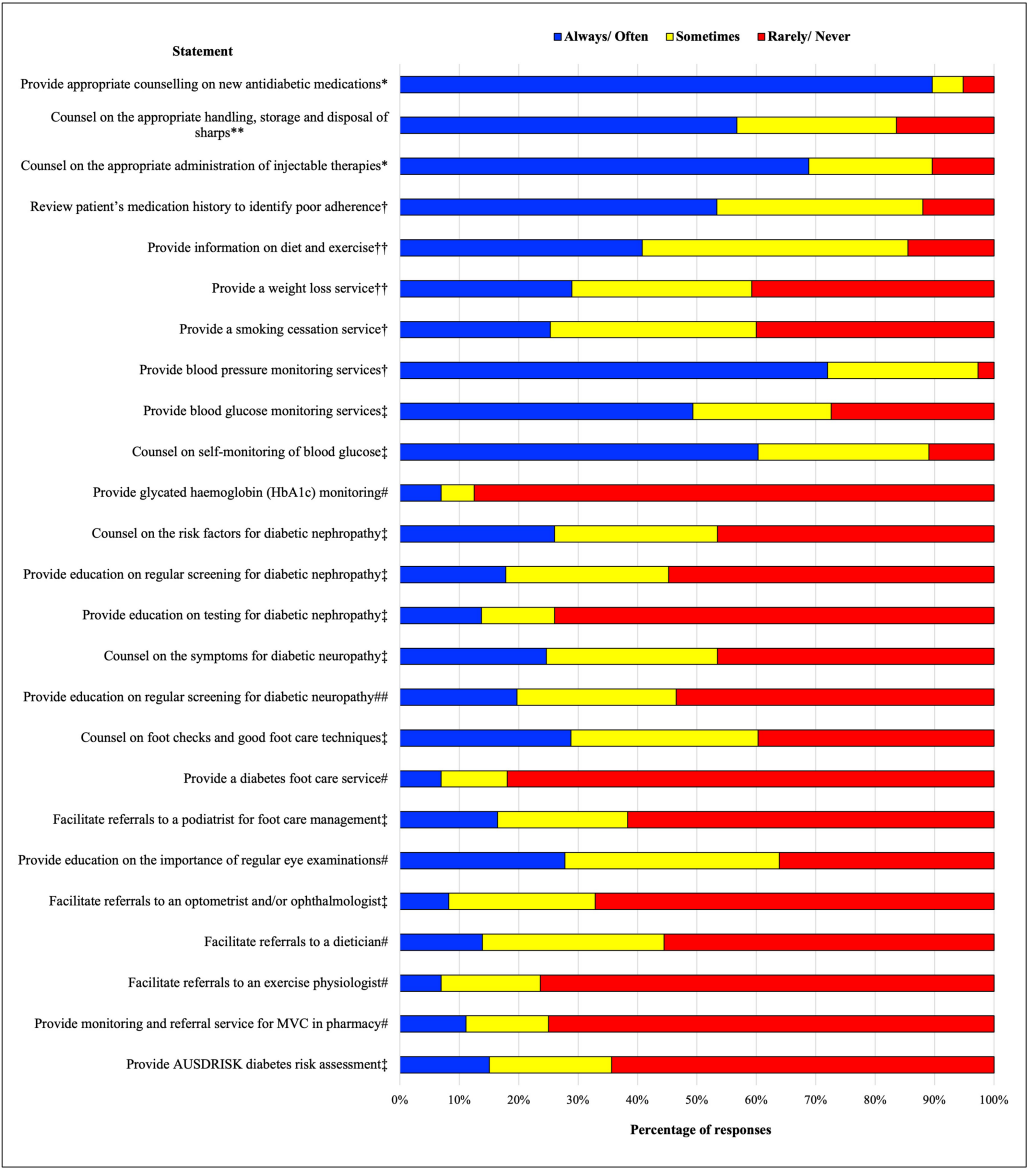
Frequency of provision of professional pharmacy services for the management of type 2 diabetes in Australian community pharmacies. T2DM, type 2 diabetes; MVC, microvascular complications; AUSDRISK, Australian Type 2 Diabetes Risk Assessment Tool. **n = 67; ##n = 71; #n = 72; ‡n = 73; †n = 75; ††n = 76; *n = 77.

In addition to the current professional services available in community pharmacies for people living with type 2 diabetes (Fig. 1), respondents reported conducting government-subsidised medication management services such as MedsCheck and Diabetes MedsCheck, and annual health checks; and providing dose administration aids (DAAs), sleep apnoea services, and education with diabetes educators. Respondents reported counselling on different aspect of microvascular complication management with varying frequency (*n* = 73). This included the signs and symptoms of DKD, diabetic retinopathy and neuropathy, regular screening for these complications, and referrals to the appropriate health professionals for monitoring (75% did not provide counselling). Interestingly, 11% reported always providing a monitoring and referral service for microvascular complications, with another 14% providing this service on occasion (*n* = 72). The least common service provided by pharmacists was formal referrals to allied health professionals. This may be due to the lack of a formal referral pathway between pharmacists and doctors. The most common service was reported to be counselling on new medicines for diabetes. This was largely anticipated, as counselling of new medicines is considered a standard practice of pharmacists in Australia. Responses to the Likert scale for part I of the questionnaire are reported in Fig. 1.

### Pharmacist attitudes to a novel screening, monitoring and referral service for microvascular complications associated with type 2 diabetes in Australian community pharmacy

**Figure 2:**
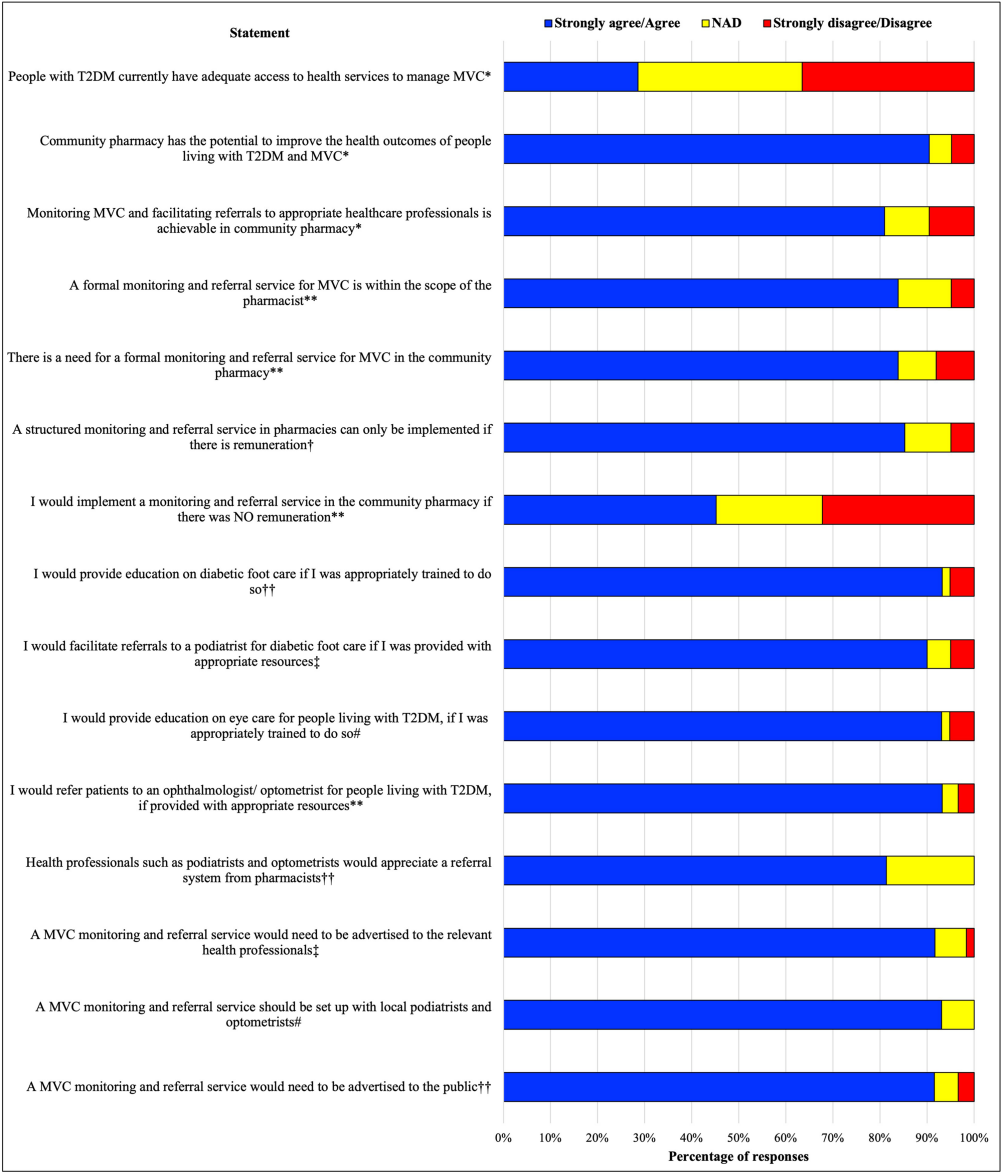
Pharmacist attitudes to a novel screening, monitoring and referral service for microvascular complications associated with type 2 diabetes in Australian community pharmacy. T2DM, type 2 diabetes; MVC, microvascular complications. #n = 58; ††n = 59; ‡n = 60; †n = 61; **n = 62; *n = 63.

The majority of respondents agreed a monitoring and referral service for microvascular complications is within the scope of practice of the pharmacist (84%, *n* = 62), is feasible in the community pharmacy (81%, *n* = 63) and identified a need for this service in the community (84%, *n* = 62). Almost all respondents (>90%, *n* = 59) would implement and provide a monitoring and referral services if provided with appropriate training and resources to liaise and refer to local allied health professionals. There was high level of agreement regarding the need for advertising to the public and collaboration with general practitioners (GPs) and health practitioners; 92% (*n* = 59) and 93% (*n* = 60), respectively (Fig. 2). The majority of respondents preferred generating a formal referral letter from an existing template to providing verbal or handwritten referrals. There were differing opinions regarding expanding pharmacists’ scope in this area. One participant suggested time restraints to appropriately provide this service, and that accredited pharmacists providing government-funded Home Medicines Reviews (HMRs) or Residential Medication Management Reviews (RMMRs) and general practice pharmacists are better situated to provide this care. Another respondent argued that *“just because pharmacy is open all the time, doesn’t mean all jobs should fall to them”*.

### Pharmacist training and continuing professional development

**Figure 3:**
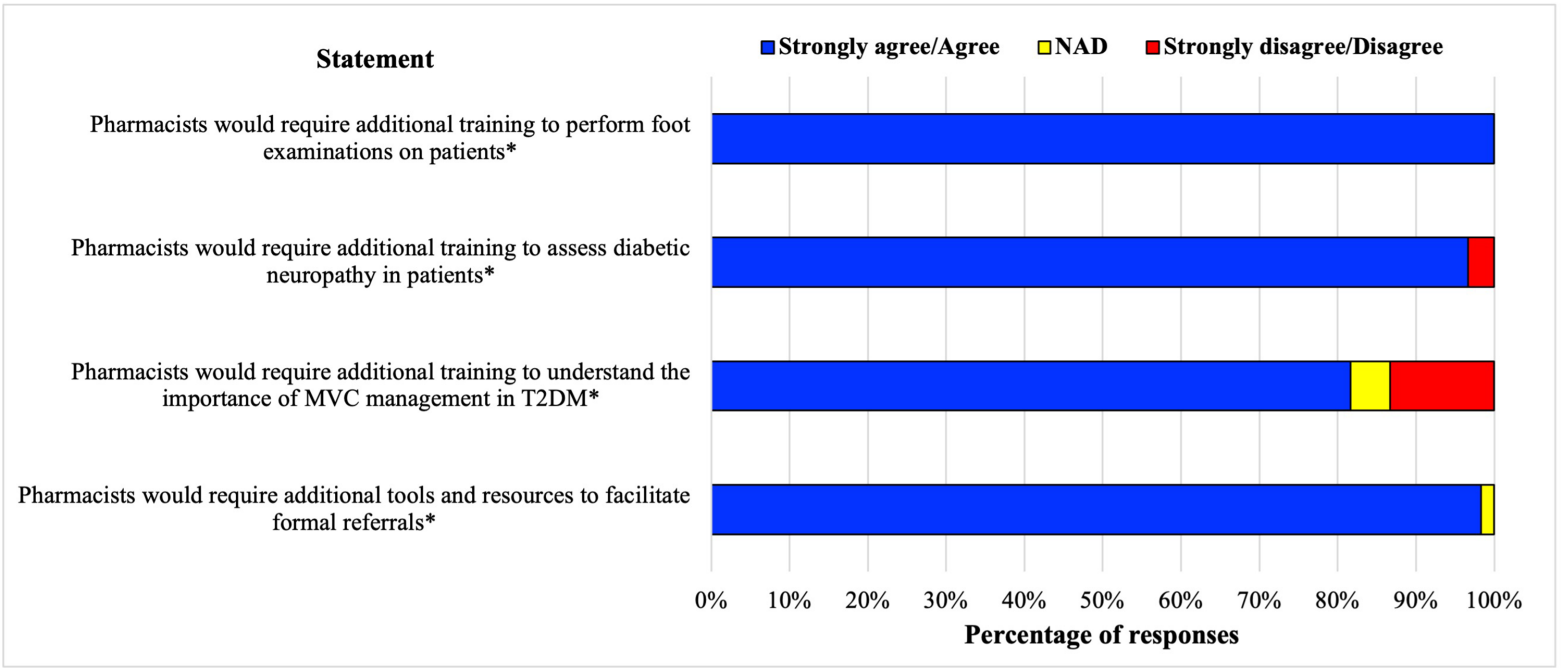
Pharmacist knowledge and training/resource requirements for implementing a structured monitoring and referral service in Australian community pharmacy. T2DM, type 2 diabetes; MVC, microvascular complications. *n = 60.

**Figure 4:**
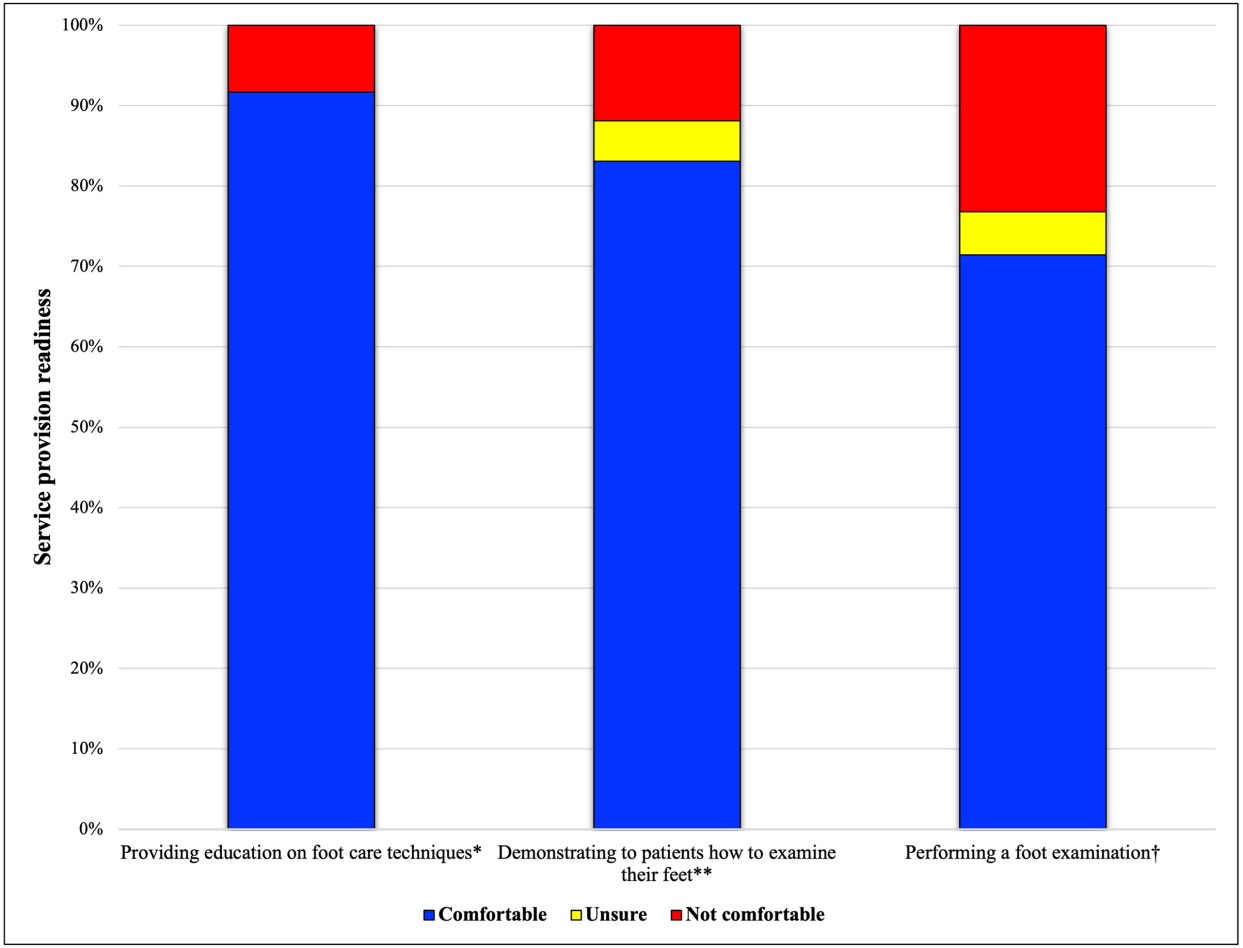
Respondents’ perceived readiness to provide services after comprehensive training. †n = 56; **n = 59; *n = 60.

All respondents agreed pharmacists required additional training to perform foot examinations if this was to be included in the screening, monitoring and referral service (100%, *n* = 60), and most respondents agreed further training and resources were required to understand microvascular complications, assess diabetic neuropathy (97%, *n* = 60), and facilitate formal referrals to health practitioners (98%, *n* = 60) (Fig. 3). The preferred mode of training delivery was combined online modules and workshops (73.8%, *n* = 62), with workshop only and online module only being the second and third choice; at 16.4% and 8.2%, respectively. Only 1.6% believed pharmacists required no additional training. It was suggested that continuing professional development modules on microvascular complications should be developed, and refresher workshops for this service made available if substantial time had passed since the initial workshop, with a variety of training delivery methods and modes. Practical training was acknowledged as an opportunity to develop technique and/or approach. Figure 4 shows pharmacists’ self-perceived readiness to provide different levels of service after comprehensive training. Over 90% of respondents stated that they would be comfortable providing education for foot self-care behaviours but only 72% would be comfortable performing foot examinations.

### Barriers to service implementation

**Figure 5:**
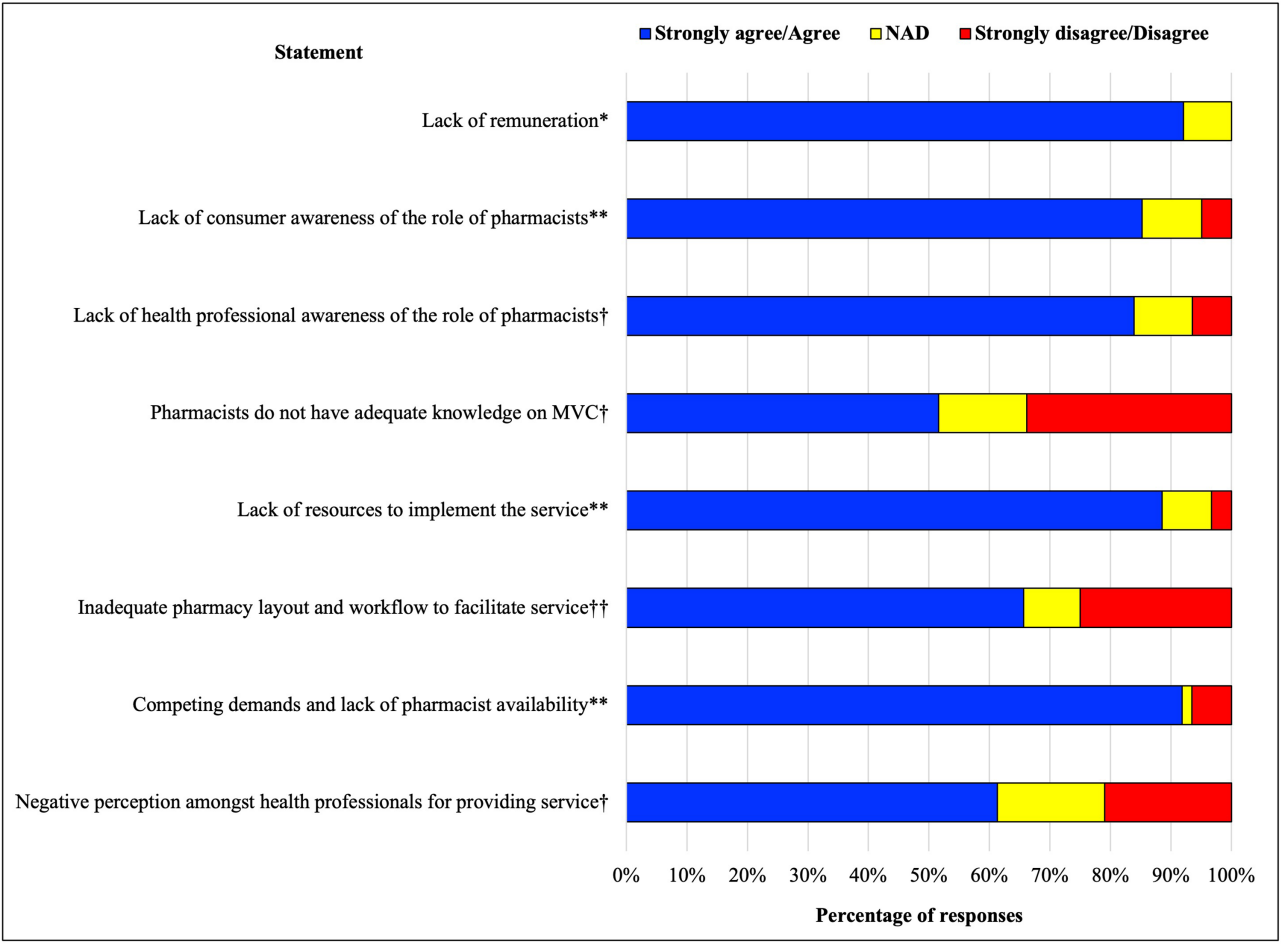
Barriers to implementing a new microvascular complication service. MVC, microvascular complications. **n = 61; †n = 62; *n = 63; ††n = 64.

Most respondents agreed that the lack of remuneration was a major barrier to implementing a structured monitoring and referral system (92%, *n* = 63), especially with the competing demands and lack of pharmacist availability (Fig. 5). Respondents also believed a lack of awareness of the role of the pharmacist in the management of type 2 diabetes and its associated complications by patients, GPs and other health professionals to be a barrier (85%, *n* = 62). One respondent commented that the role of the pharmacist is not necessarily outlined in the diabetes multidisciplinary team in the community, so pharmacist integration into the team would require a collaborative approach. Approximately half (52%, *n* = 62) of the respondents believed pharmacists do not possess the required knowledge to appropriately manage microvascular complications, especially if the pharmacist is not a diabetes educator. An inadequate pharmacy layout and lack of resources were considered additional barriers. Pharmacists in general practice were suggested to conduct this service. Furthermore, respondents commented patient time could be a barrier, and that patients could be reluctant to pay for this service in community pharmacy.

### Service delivery models

The majority of respondents believed the service would be more efficient with automatic reminders and automatic referrals for check-ups with podiatrists and optometrists/ophthalmologists if incorporated into current dispensing software (77.0%, *n* = 61). Respondents preferred to create a referral from a template on a separate platform or software. There were a range of different responses provided by pharmacists in relation to who should pay for this service (Fig. S1). The estimated time to perform screening, monitoring and referral service ranged from 10 to 60 min, with most respondents estimating 15 to 45 min to complete the service (*n* = 29).

## Discussion

This is the first comprehensive study investigating the potential for a screening, monitoring and referral service for microvascular complications associated with type 2 diabetes in Australian community pharmacies. Overall, respondents were positive about the implementation of a novel screening, monitoring and referral service. It was acknowledged that this could only be accomplished if adequate remuneration and specific training were available for pharmacists. The proposed service extends beyond the current roles of pharmacists in diabetes management in community pharmacies and addresses the gap with microvascular complication management. Current professional pharmacy services provide support to people who live with type 2 diabetes through diabetes medication reviews, BGL and HbA1c testing, weight loss programs and blood pressure services, however there is minimal specific attention to microvascular complication management. There is currently no requirement for Australian community pharmacists to undertake specific credentialing to provide basic diabetes services, with medication provision, counselling, screening and monitoring of HbA1c and BGL covered during university training and considered within the scope of practice of a generally registered pharmacist. The majority of participants, however, agreed that they would need additional education and training to provide any of the proposed microvascular complication-focussed services.

Respondents believed that a screening, monitoring and referral service for microvascular complications is within pharmacists’ scope of practice. A need for this service was also identified, however the level of service requires further exploration. Respondents expressed some interest in providing a range of services based on those trialled internationally. These international pharmacist-led foot care interventions have proven successful in early intervention and management and potentially reducing the overall cost of diabetes-related foot complications (Soprovich et al., 2019). Pharmacist intervention ranged from reminders about foot self-care behaviours with each visit to the pharmacy (Abduelkarem & Sackville, 2009; Jahangard-Rafsanjani et al., 2015; Mehuys et al., 2008) to promoting foot examinations by health professionals (Bluml et al., 2014; Fera, Bluml & Ellis, 2009; Pinto, Bechtol & Partha, 2012; Poulose et al., 2015). The foot examinations were either performed, or the referral made to a podiatrist, by a pharmacist. These studies showed significant improvements in foot care behaviours, however it was noted that ongoing reminders to patients were required to support continued progress and behaviour changes (Abduelkarem & Sackville, 2009). Furthermore, a Canadian case study performing point-of-care nerve conduction foot examinations in a community pharmacy found 57% of the participants had mild or moderate nerve conduction abnormalities, despite 46% of participants reporting no signs or symptoms of diabetic neuropathy (Poulose et al., 2015). Pharmacists agreed it was easy to perform the test and interpret the results but more importantly it was valuable in educating patients on the relationship between good glycaemic and blood pressure control and diabetic neuropathy (Poulose et al., 2015). There is no legislative limitation to providing pharmacist-led foot care interventions in Australia, therefore similar models could be adopted in Australian community pharmacies.

Unsurprisingly, remuneration was identified as the major determinant of the successful implementation and sustainability of the proposed service. Whether remuneration is from government funding, patients or private health funds, lack of remuneration for pharmacist-led services is an international problem and a major barrier in service implementation (Nordin, Hassali & Sarriff, 2017). Competing demands, pharmacist time constraints, especially during the COVID-19 pandemic and other natural disaster or emergency situations and maintaining relationships with other health professionals were also perceived as leading barriers for the implementation of this service. Allied health professionals may have negative perceptions about this service, so it is imperative effective collaboration with these professionals is achieved. These barriers may have contributed to some pharmacists’ lack of readiness to embrace the new service. Interdisciplinary education and raising awareness of allied health professionals about the roles and scope of pharmacist practice may address this issue. Based on the findings of this study, a monitoring and referral service focussing on diabetic foot care education, reminders on self-foot checks with monthly supply of medication and facilitating formal referrals to appropriate health professionals may be more readily implemented in community pharmacies than a more complex service. Services involving pharmacists performing foot examinations might be considered an advanced service and may be less likely to be implemented in pharmacies. Due to the ever-changing environment and expectations of community pharmacy, it may not be feasible for pharmacists to perform foot examinations with current time restraints and without appropriate remuneration (Nordin, Hassali & Sarriff, 2017). However, the incorporation of a novel screening, monitoring and referral service into an existing diabetes-related service could potentially allow for service implementation (Maidment et al., 2021).

There are a few barriers to service implementation in community pharmacies. Software requirements for generating timely referrals were identified as a barrier to service implementation. Different types of referrals are available and range from basic verbal referrals to more structured formal referral letters (Sim et al., 2020). Automatic reminders and referrals for check-ups with podiatrists and optometrists incorporated into dispensing software in community pharmacies was the preferred method of software integration. It is essential to have established communication between health professionals for the prevention and management of microvascular complications (Sim et al., 2020). Unlike GPs, pharmacists facilitating formal referrals to healthcare professionals would not be government-subsidised, therefore patients would have to be willing to pay privately. Consistent review by podiatrists, optometrists/ophthalmologists, exercise physiologists and dieticians will assist in early detection of diabetic foot disease and diabetic retinopathy, as well as ensuring lifestyle modifications are adhered to (Medical Advisory Secretariat, 2009). People living with type 2 diabetes generally see their local pharmacist once a month for medication supply. There is an opportunity to further utilise pharmacists to provide education on microvascular complications and facilitate timely referrals to podiatrists and optometrists/ophthalmologists as per national guidelines. This integration of pharmacists within multidisciplinary teams is vital in the successful management of microvascular complications associated with type 2 diabetes (Al AdAwi et al., 2020). If pharmacists are incorporated within multidisciplinary teams, there needs to be clear role delineation between pharmacists and other healthcare professionals. There are legislative barriers that limit pharmacist monitoring and reporting of DKD. Australian community pharmacists are unable to initiate or submit pathology requests and therefore cannot request DKD monitoring tests. Pharmacists can provide medication reviews to optimise medication management for DKD, however a formalised, structured screening, monitoring and referral service would provide pharmacists with the resources to generate referrals to GPs to further monitor DKD, including the requests for blood and urine tests.

There are some limitations to this research. Firstly, there was a low response rate. The questionnaire was released and distributed during high COVID-19 caseloads, restrictions and lockdowns, especially in the eastern states of Australia, and the roll out of COVID-19 vaccinations in community pharmacy. The associated staff shortages, conflicting priorities, and burnout and stress among pharmacists (Australian Government Department of Health, 2022) may have contributed to the low response rate, and the disproportionate response from Western Australia *vs* the eastern states. More than half the respondents were under 30 years of age and registered for less than 5 years, and almost two thirds were practicing in Western Australia, and therefore not representative of Australian community pharmacists as a whole (Pharmacy Board of Australia, 2020), meaning the results may not accurately represent the views of all community pharmacists. Despite the limitations in sample size and generalisability due to reduced capacity of pharmacists during the COVID-19 pandemic, the questionnaire demonstrates pharmacist willingness to provide this service even during COVID-19. The pandemic demonstrated the flaws in the healthcare system and showed healthcare at breaking point. There is a considerable push to strengthen primary healthcare and considering people living with diabetes reside in the community, pharmacists providing this service will address existing management gaps. It is worth noting that early career pharmacists may be more eager to implement extended professional services however they are also less likely to be in positions to create change short-term; they will however be future leaders in the industry. Furthermore, this questionnaire only addressed the pharmacist’s perspective. Future work will explore the perspectives of consumers, as potential service users, on diabetes-related services, and specifically the proposed service; as well as the perspectives of other health professionals. Other future studies will involve creating and validating educational content and resources to upskill pharmacists, in preparation for a large scale implementation trial of pharmacist-led screening for diabetes-related microvascular complications. It is important to explore the association between demographics and pharmacist attitudes as part of the implementation process. This will provide insights as to where and how this professional service would best ‘fit’ in practice.

## Conclusions

Overall, pharmacists were in support of implementing a novel screening, monitoring and referral service for microvascular complications associated with type 2 diabetes, after completing specific training and if appropriate remuneration is available. Whilst there was some uncertainty about pharmacists performing foot examinations in the pharmacy, respondents were comfortable in providing further education on microvascular complications and facilitating timely referrals to the appropriate health professional when required. There is an opportunity to utilise the accessibility of pharmacists in the management of microvascular complications to ensure early detection of such complications and improve the quality of life in people living with type 2 diabetes, although the development and implementation of the service needs to be more fully explored.

## Supplemental Information

10.7717/peerj.14849/supp-1 Supplemental Information 1 Survey.

10.7717/peerj.14849/supp-2 Supplemental Information 2 Sources of payment for service.

10.7717/peerj.14849/supp-3 Supplemental Information 3 Community pharmacists’ role in the management of type 2 diabetes and related microvascular complications: A nationwide survey in Australia questionnaire responses.

## Additional Information and Declarations

### Data Availability

The following information was supplied regarding data availability: The raw data and deidentified questionnaire responses are available in the Supplemental Files.
