# Supplementary material for: Developing community pharmacists’ role in the management of type 2 diabetes and related microvascular complications: a nationwide survey in Australia
Source: PeerJ. 2023 Feb 16;11:e14849. doi: 10.7717/peerj.14849 (PMC9939021; doi:10.7717/peerj.14849)
Supplement: Supplemental Information 1 [file peerj-11-14849-s001.pdf]

Participant Information and Consent

Participant Information Statement

HREC Project Number	74225
Project Title:	Community pharmacists' role in the management of type 2 diabetes and its microvascular complications: A nationwide survey
Chief Investigator:	Dr Leanne Chalmers, Senior Lecturer, Curtin Medical School
Other Investigators:	Dr Tin Fei Sim, Senior Lecturer; Professor Graham Hillis, Adjunct Professor; Curtin Medical School

Student researcher:	Louise Woodhams
Version Number:	2
Version Date:	18/03/2021

What is this study about?

This study aims to investigate the current role of pharmacists in type 2 diabetes management and in the management and monitoring of microvascular complications in the primary care community setting. Microvascular complications include diabetic nephropathy, retinopathy and neuropathy. The current practice of pharmacists will be examined, as well as the need for further education in monitoring and referrals for microvascular complications associated with type 2 diabetes. This questionnaire will also assess the readiness of pharmacists in implementing this service and the current barriers faced by pharmacists.

The objectives of this questionnaire include:

1. Investigate the current practice of community pharmacists in diabetes management,

2. Investigate the barriers in implementing a microvascular complication management service in a community pharmacy setting,
3. Explore community pharmacists’ attitudes and perspectives on the implementation of microvascular complication management services,
4. Identify the need for continued professional development and the development of future training, and
5. Explore potential service delivery models.

Who is doing the study?

This project will be conducted by Louise Woodhams from the Curtin Medical School at Curtin University as part of a Doctor of Philosophy (Pharmacy) degree. This project is under the supervision of Dr Leanne Chalmers, Dr Tin Fei Sim and Adjunct Professor Graham Hillis from Curtin Medical School, Curtin University.

What does participation in the research entail?

Participation in this study involves completing a questionnaire on the current roles of pharmacists in diabetes management and the readiness of pharmacists to implement microvascular complications monitoring and referral services. While it is not mandatory to complete every question, responses that are detailed and comprehensive will strengthen the results and help to positively impact the profession in the future. There will be a

section at the end for further comments. The questionnaire should take no longer than X minutes to complete.

Are there any benefits to participating in the study?

By participating in this questionnaire, participants will be invited to enter a prize draw. Other benefits include potential expansion of scope of practice in diabetes management.

Are there any risk, side effects, discomforts or inconveniences from being involved in the study?

Whilst all efforts have been made to ensure the identity of participants remains anonymous, there is a possibility that participants may be identified from responses in the questionnaire. Information retrieved from the questionnaire that has the ability to identify participants will not be reproduced in any format.

Who will have access to my information?

This study complies with the Privacy Act 1988 and any information retrieved from the study that is personal and/or identifiable will remain confidential and will not be disclosed to third parties or reported. The questionnaire does not ask for any identifiable information.

The research team will have access to the information collected from the study. In the event of an audit or investigation, staff from the Curtin University Office or Research and Development will also have access to information collected from the study.
All electronic data will be stored on password-protected servers and hard, as per the data management plan. Data will be stored for seven years post-study. The data retrieved from this questionnaire will be used as part of a PhD thesis and for publication. De-identified quotes from the questionnaire may be reported in publications and/or this thesis to illustrate themes identified.

Will you tell me the results of the study?
At the completion of the study, the results of the study will be available online in a publication.

Do I have to take part in the study?
Participation in this study is completely voluntary. Consent is implied for participation and for the use of information when the questionnaire is completed and submitted. This questionnaire is completely anonymous and will not require participants to disclose personal or identifiable information. Withdrawal of data after submission of questionnaire responses is not possible as submitted information is unidentifiable.

What happens next and who can I contact about the study?
Should you require any further information about this study, please contact the research team: Louise Woodhams (louise.woodhams@postgrad.curtin.edu.au), Leanne Chalmers (leanne.chalmers@curtin.edu.au) or Tin Fei Sim (t.sim@curtin.edu.au).

If you wish to participate in this study, please click on the checkbox below to indicate you have read and understood the information provided in the participant information sheet and consent to be involved. The questionnaire will begin on the next page.

This study was approved by Curtin University Human Research Ethics Committee (HREC number XXXX). Should you wish to discuss any matters concerning the conduct of the study, your rights as a participant, or you wish to make a confidential complaint, you may contact the Ethics Officer on (08) 266 9223 or email hrec@curtin.edu.au.

I consent to participate in this study.

Part I: Current practice of community pharmacists in diabetes management.

1. Please answer the following statements below, regarding your practice as a pharmacist in how you currently manage type 2 diabetes (T2DM) and its associated complications in the pharmacy.

Never: I do NOT provide this service to patients
Rarely: I provide this service approximately 25% of the time to patients with T2DM
Sometimes: I provide this service approximately 50% of the time to patients with T2DM
Often: I provide this service approximately 75% of the time to patients with T2DM
Always: I provide this service approximately 100% of the time to patients with T2DM

	Never	Rarely	Sometimes	Often	Always
A. Provide appropriate counselling on new antidiabetic medications.	○	○	○	○	○

	Never	Rarely	Sometimes	Often	Always
B. Counsel on the appropriate handling, storage and disposal of sharps (e.g. syringes, needles, lancets).	○	○	○	○	○
C. Counsel on the appropriate administration of injectable therapies.	○	○	○	○	○
D. Review patient's medication history to identify poor adherence.	○	○	○	○	○
E. Provide information on diet and exercise as it relates to diabetes management.	○	○	○	○	○
F. Provide a weight loss service in the pharmacy for diabetes management, including supply of weight loss products.	○	○	○	○	○
G. Provide a smoking cessation service in the pharmacy for diabetes management.	○	○	○	○	○
	Never	Rarely	Sometimes	Often	Always

	Never	Rarely	Sometimes	Often	Always
H. Provide blood pressure monitoring services in the pharmacy.	○	○	○	○	○
I. Provide blood glucose monitoring services in the pharmacy.	○	○	○	○	○
J. Counsel on self-monitoring of blood glucose.	○	○	○	○	○
K. Provide glycosylated haemoglobin (HbA1c) monitoring in the pharmacy.	○	○	○	○	○
L. Counsel on the risk factors for diabetic nephropathy.	○	○	○	○	○
M. Provide education on the importance of regular screening for nephropathy.	○	○	○	○	○
N. Provide education on testing for diabetic nephropathy, such as the type of test, testing frequency and testing locations.	○	○	○	○	○

	Never	Rarely	Sometimes	Often	Always
	Never	Rarely	Sometimes	Often	Always
O. Counsel on the symptoms of diabetic neuropathy (e.g. neuropathic pain, paresthesia, numbness, formication, etc).	○	○	○	○	○
P. Provide education on the importance of regular screening for diabetic neuropathy.	○	○	○	○	○
Q. Counsel on foot checks and good foot care techniques.	○	○	○	○	○
R. Provide a diabetes foot care service in the pharmacy.	○	○	○	○	○
S. Facilitate referrals to a podiatrist for foot care management in diabetes.	○	○	○	○	○
T. Provide education on the importance of regular eye examinations.	○	○	○	○	○

	Never	Rarely	Sometimes	Often	Always
U. Facilitate referrals to an optometrist and/or ophthalmologist.	◯	◯	◯	◯	◯

	Never	Rarely	Sometimes	Often	Always
V. Facilitate referrals to a dietician for diabetes management.	◯	◯	◯	◯	◯
W. Facilitate referrals to an exercise physiologist for diabetes management.	◯	◯	◯	◯	◯
X. Provide monitoring and referral service for microvascular complications in the pharmacy.	◯	◯	◯	◯	◯
Y. Provide AUSDRISK diabetes risk assessment.	◯	◯	◯	◯	◯

Part I: Current practice of community pharmacists in diabetes management.

2. Do you provide any other services to your patients with T2DM that we have not covered? If so, please list them here.

Part II: Attitudes on microvascular complication management services.

Part II: Attitudes on microvascular complication management services.

3. For each of the statements below, click on the response that best characterises your opinion about the statement.

	Strongly disagree	Disagree	Unsure	Agree	Strongly agree
A. People with type 2 diabetes currently have adequate access to health services to manage microvascular complications (nephropathy, retinopathy and neuropathy).	○	○	○	○	○

	Strongly disagree	Disagree	Unsure	Agree	Strongly agree
B. Community pharmacy has the potential to improve the health outcomes of people living with diabetes and microvascular complications.	○	○	○	○	○
C. Monitoring microvascular complications and facilitating referrals to appropriate healthcare professionals is achievable in community pharmacy.	○	○	○	○	○
D. A formal monitoring and referral service for microvascular complications is within the scope of the pharmacist.	○	○	○	○	○
E. There is a need for a formal monitoring and referral service for microvascular complications in the community pharmacy.	○	○	○	○	○
	Strongly disagree	Disagree	Unsure	Agree	Strongly agree

	Strongly disagree	Disagree	Unsure	Agree	Strongly agree
F. A structured monitoring and referral service in pharmacies can only be implemented if there is remuneration.	○	○	○	○	○
G. If a structured monitoring and referral service was available for pharmacists and pharmacies, I would implement this service in the community pharmacy if there was NO remuneration.	○	○	○	○	○
H. I would provide education on diabetic foot care if I was appropriately trained to do so.	○	○	○	○	○
I. I would facilitate referrals to a podiatrist for diabetic foot care if I was provided with appropriate resources.	○	○	○	○	○
J. I would provide education on eye care for people living with T2DM, if I was appropriately trained to do so.	○	○	○	○	○
	Strongly disagree	Disagree	Unsure	Agree	Strongly agree

	Strongly disagree	Disagree	Unsure	Agree	Strongly agree
K. I would refer patients to an ophthalmologist/ optometrist for people living with T2DM, if provided with appropriate resources.	○	○	○	○	○
L. Health professionals such as podiatrists and optometrists would appreciate a referral system from pharmacists.	○	○	○	○	○
M. An in-pharmacy microvascular monitoring and referral service would need to be advertised to the relevant health professionals such as podiatrists and optometrists.	○	○	○	○	○
N. An in-pharmacy microvascular monitoring and referral service should be set up with local podiatrists and optometrists.	○	○	○	○	○
O. An in-pharmacy microvascular monitoring and referral service would need to be advertised to the public.	○	○	○	○	○

Part II: Attitudes on microvascular complication management services.

4. Please indicate to what extent you believe the following to be barriers to implementing a new microvascular complication service.

	Strongly disagree	Disagree	Unsure	Agree	Strongly agree
A. Lack of remuneration	○	○	○	○	○
B. Lack of consumer awareness of the role of pharmacists in diabetes management.	○	○	○	○	○
C. Lack of health professional awareness of the role of pharmacists in diabetes management.	○	○	○	○	○
D. Pharmacists do not have adequate knowledge on microvascular complications.	○	○	○	○	○
	Strongly disagree	Disagree	Unsure	Agree	Strongly agree

	Strongly disagree	Disagree	Unsure	Agree	Strongly agree
E. Lack of resources to implement the service (information for pharmacists, pharmacy software, information to give to patients, etc)	○	○	○	○	○
F. Inadequate pharmacy layout and workflow to facilitate service provision.	○	○	○	○	○
G. Competing demands and lack of pharmacist availability.	○	○	○	○	○
H. Negative perception among other health professionals towards pharmacists providing the service.	○	○	○	○	○

Part II: Attitudes on microvascular complication management services.

5. Please list any other barriers you believe will influence implementing this service.

Part III: Knowledge, continuing professional development and training.

Part III: Knowledge, continuing professional development and training.

6. For each of the statements below, click on the response that best characterises your opinion about the statement.

	Strongly disagree	Disagree	Unsure	Agree	Strongly agree
A. People living with type 2 diabetes are required to have foot examinations every 6 months.	○	○	○	○	○
B. People living with type 2 diabetes are required to have eye examinations every 2 years.	○	○	○	○	○

	Strongly disagree	Disagree	Unsure	Agree	Strongly agree
C. 1 in 10 people living with type 2 diabetes will develop diabetic kidney disease at some point in their life.	○	○	○	○	○
D. Microvascular complications develop from poorly controlled blood glucose and blood pressure.	○	○	○	○	○
E. Diabetic neuropathy mostly causes nerve damage in the legs and feet.	○	○	○	○	○
F. Pharmacists would require additional training to perform foot examinations on patients.	○	○	○	○	○
G. Pharmacists would require additional training to assess diabetic neuropathy in patients.	○	○	○	○	○
H. Pharmacists would require additional training to understand the importance of microvascular complication management in people living with type 2 diabetes.	○	○	○	○	○

	Strongly disagree	Disagree	Unsure	Agree	Strongly agree
I. Pharmacists would require additional tools and resources to facilitate formal referrals.	○	○	○	○	○

Part III: Knowledge, continuing professional development and training.

7. Please compare the different training modes and select which training method you would prefer.

	**Method A**	**Method B**	**Method C**	**Metho D**
Format	Completely online	Workshop only	Online module and workshop	No trainin[illegible]
Examinations and referral points	Video of foot examinations by a podiatrist.	In-person demonstration of foot examinations	Video and in-person demonstrations of foot	

	Video of eye referral points by optometrist.	by a podiatrist. In-person demonstration of eye referral points by optometrist.	examinations by podiatrist and eye referral points by optometrist.	
Tools and resources	Monitoring and referral tool resources available.	Monitoring and referral tool resources available.	Monitoring and referral tool resources available.	

Method A

Method B

Method C

Method D

Part III: Knowledge, continuing professional development and training.

8. Please add any additional comments here, regarding preferred method of training.

Part IV: Potential service delivery models.

Part IV: Potential service delivery models.

9. Question 9 relates to software requirements for a screening, monitoring and referral system for microvascular complication T2DM management service. Please choose from the following options the software design that you believe most suitable.

- Service runs on a separate platform/ software.
- Service is incorporated into current dispensing software.
- No software is required for this service

Part IV: Potential service delivery models.

10a. Please indicate on the scale below how comfortable you would feel providing the service listed below (after comprehensive training, where necessary).

Service 1: Providing education on foot care techniques.

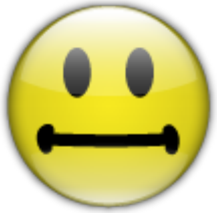

Part IV: Potential service delivery models.

10b. Please indicate on the scales below how comfortable you would feel providing the service listed below (after comprehensive training, where necessary).

Service 2: Demonstrating to patients how to examine their feet.

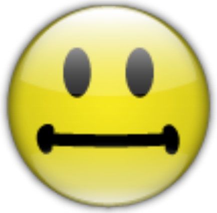

Part IV: Potential service delivery models.

10c. Please indicate on the scale below how comfortable you would feel providing the service listed below (after comprehensive training, where necessary).

Service 3: Performing a foot examination.

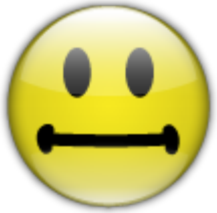

Part IV: Potential service delivery models.

11. Out of the two options for a service delivery model outlined below, please rank your preference, with 1 = first choice and 2 = last choice.

Service relies on pharmacist to remember to educate and counsel on regular follow-ups with podiatrist and optometrist.

Service includes automatic reminders for foot, eye and blood tests whilst dispensing prescriptions.

Service is linked to existing services in community pharmacy, such as Diabetes Medschecks and health checks/ screening.

Service is provided on an ad hoc basis, when monitoring needs are identified.

There is no need for a monitoring and referral service.

Part IV: Potential service delivery models.

12. Below are examples of referral services. Please rank these services from first choice to last choice.

1 = you would most likely want to do

4 = you would least likely want to do.

Verbal referral from pharmacist.

Hand-written referral by pharmacist.

Referral created by pharmacist from template on separate platform/ software.

Automated referral process whilst dispensing.

Part IV: Potential service delivery models.

13. Imagine a screening, monitoring and referral service for microvascular complications exists in your pharmacy. The process and steps for this service are shown below:

Step 1: Automated reminder for annual foot check appears whilst dispensing patient's prescriptions.
Step 2: The pharmacist assesses the need for the service.
Step 3: The pharmacist provides written and verbal counselling on foot care techniques and the reasons why this is important.

Step 4: The pharmacist provides a demonstration on how to perform a foot examination, so that the patient can perform regular checks at home.
Step 5: The pharmacist performs a foot examination for the patient.
Step 6: An automated referral to the local podiatrist is generated, emailed to the practitioner and printed for the patient.

Based on this information, how long (in minutes) do you think this service would take to complete?

Part IV: Potential service delivery models.

14. Who do you think should pay for this service described above?

Part V: Demographics

Part V: Demographics

15. What is your gender?

Male

Female

Other

Prefer not to say

Part V: Demographics

16. What is your age?

<30 years old

30-40 years old

41-50 years old

51-60 years old

>60 years

Part V: Demographics

17. How long have you been a registered pharmacist in Australia?

<5 years

5-10 years

11-15 years

16-20 years

>20 years

Part V: Demographics

18. Which of the following best describes your role in the community pharmacy? If you work in multiple pharmacies, please state your position in your primary workplace.

Sole proprietor

Partner proprietor

Pharmacist in charge

Employee pharmacist

Other (please specify)

Part V: Demographics

19. Which of the following best describes the setting of the community pharmacy?

- City centre
- Shopping strip
- Shopping centre
- Isolated
- Next to medical centre
- Other (please specify)

Part V: Demographics

20. Which of the following best describes the size of operation of this pharmacy?

- Small (turnover <$2 million per annum)
- Large (turnover >$2 million per annum)

Unsure

Part V: Demographics

21. Which state/ territory do you work in?

Australian Capital Territory (ACT)
New South Wales (NSW)
Northern Territory (NT)
Queensland (QLD)
South Australia (SA)
Tasmania (TAS)
Victoria (VIC)
Western Australia (WA)

Part V: Demographics

22. Which of the following best describes the location of the community pharmacy?

City

Suburb

Rural

Remote

Part V: Demographics

23. Which of the following best describes the location of this pharmacy in relation to the nearest medical practice?

Co-located

<100 metres

101-500 metres

501 metres - 1 kilometre

>1 kilometre

Part V: Demographics

24. What is your highest level of pharmacy education?

Bachelor of Pharmacy

Bachelor of Pharmacy (Hons)

Master of Pharmacy

PharmD

PhD

Part V: Demographics

25. Are you an accredited pharmacist?

Yes, accredited pharmacist

Yes, undergoing accreditation

No, not an accredited pharmacist or undergoing accreditation

Part V: Demographics

26. Are you an accredited diabetes educator?

Yes, accredited diabetes educator

No, but undergoing accreditation

No, not an accredited diabetes educator or undergoing accreditation

This is the end of the survey. Clicking on the arrow below will submit your response.

Powered by Qualtrics
