# Supplementary figures and images for: Developing community pharmacists’ role in the management of type 2 diabetes and related microvascular complications: a nationwide survey in Australia
Source: PeerJ. 2023 Feb 16;11:e14849. doi: 10.7717/peerj.14849 (PMC9939021; doi:10.7717/peerj.14849)

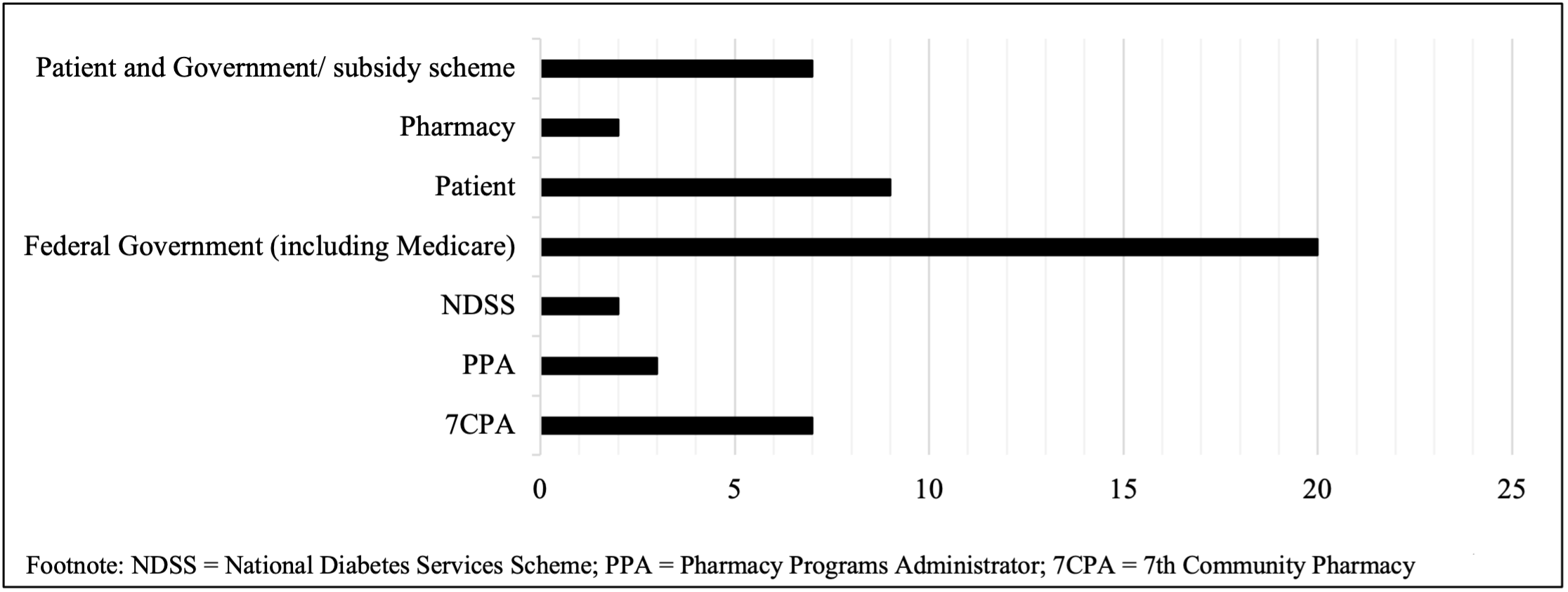

Supplement: Supplemental Information 2 [file peerj-11-14849-s002.png]
